# Orphan high field superconductivity in non-superconducting uranium ditelluride

**DOI:** 10.1038/s41467-024-47090-1

**Published:** 2024-04-20

**Authors:** Corey E. Frank, Sylvia K. Lewin, Gicela Saucedo Salas, Peter Czajka, Ian M. Hayes, Hyeok Yoon, Tristin Metz, Johnpierre Paglione, John Singleton, Nicholas P. Butch

**Affiliations:** 1 NIST Center for Neutron Research, National Institute of Standards and Technology, Gaithersburg, MD USA; 2 Maryland Quantum Materials Center, Department of Physics, University of Maryland, College Park, MD USA; 3 Canadian Institute for Advanced Research, Toronto, ON M5G 1Z8 Canada; 4 National High Magnetic Field Laboratory, Los Alamos National Laboratory, Los Alamos, NM USA

**Keywords:** Superconducting properties and materials, Topological matter

## Abstract

Reentrant superconductivity is an uncommon phenomenon in which the destructive effects of magnetic field on superconductivity are mitigated, allowing a zero-resistance state to survive under conditions that would otherwise destroy it. Typically, the reentrant superconducting region derives from a zero-field parent superconducting phase. Here, we show that in UTe_2_ crystals extreme applied magnetic fields give rise to an unprecedented high-field superconductor that lacks a zero-field antecedent. This high-field orphan superconductivity exists at angles offset between 29^o^ and 42^o^ from the crystallographic *b* to *c* axes with applied fields between 37 T and 52 T. The stability of field-induced orphan superconductivity presented in this work defies both empirical precedent and theoretical explanation and demonstrates that high-field superconductivity can exist in an otherwise non-superconducting material.

## Abstract (web summary)

In addition to its low-field superconducting state, UTe_2_ features a re-entrant superconducting state when high magnetic fields are applied at a particular range of angles. Here, the authors demonstrate that the high-field re-entrant superconducting state survives even when the low-field superconducting state is destroyed by disorder.

## Introduction

Applied magnetic fields destabilize and eventually destroy superconductivity by breaking up the constituent paired electrons. In most cases, this occurs through the effect of orbital pair-breaking, a condition wherein magnetic flux cores overlap. A competing pair-breaking effect occurs at the Pauli limit, the typically higher magnetic field at which Zeeman splitting destabilizes spin anti-aligned Cooper pairs^1^. In uranium ditelluride (UTe_2_) crystals that exhibit a low-field superconducting transition, however, superconductivity survives to fields that well exceed the Pauli limit, due to the occurrence of unconventional spin-triplet superconductivity^2,3^. When the magnetic field is applied along the crystallographic *b* axis, superconductivity survives to a remarkably large magnetic field value of 35 T, limited only by a first-order metamagnetic transition–a discontinuity in the magnetization– at *H*_*m*_. However, if the magnetic field is tilted between a range of angles 20^o^−40^o^ from the crystallographic *b*-axis towards the *c-*axis^4,5^, superconductivity returns for fields greater than 40 T, persisting to approximately 70 T. The focus of this work is the relationship between this very high-field reentrant superconductivity (SC_FP_) and the low-field phases (SC_1_ and SC_2_); SC_1_ is generally assumed to be the primary, or “parent,” superconducting phase.

The properties of the lower-field superconductivities in UTe_2_ have been extensively studied, but the symmetries of the superconducting order parameter(s) have yet to be unambiguously determined^6–8^. From specific heat capacity and optical Kerr effect measurements, it was inferred that superconductivity in the lowest-field phase, SC_1_, can be described by a chiral, time-reversal symmetry breaking, multi-component order parameter^6^. More recent investigations call into question the existence of a two-component order parameter and whether the state intrinsically breaks time reversal symmetry^9–11^. Evidence for a low-field point node gap structure is robust^12–14^, but has recently been questioned^15^. Experimental evidence suggests that applied fields oriented along the *b* axis induce transitions between multiple superconducting phases^16^, though the pairing states of and sample-dependent boundaries between these phases remain unclear^17,18^.

The dominant feature in the high-field UTe_2_ phase diagram when the field is nearly parallel to the *b* axis is the metamagnetic transition from either SC_2_ into a field-polarized normal state at applied field *H*_*m*_. The curving *H*_*m*_. boundary line has a minimum of about 35 T when the field is perfectly oriented along *b* and increases smoothly as the field is rotated towards one of the other crystallographic axes. The most extraordinary aspect of this phase diagram is SC_FP_, a pocket of zero resistance emerging at field orientations 20^o^–40^o^ between *b* and *c*. The lower boundary of SC_FP_ follows *H*_*m*_, which at these angles occurs at approximately 40 T^4,5^.

Due to the unprecedentedly high fields required to stabilize the SC_FP_ superconducting phase, determining its pairing symmetry presents an even greater challenge than those of SC_1_ and SC_2_, and explorations have been limited despite plain fundamental interest^3,19–25^. It is difficult to concretely establish the nature of the relationship between the lower field superconducting phases and SC_FP_ as there are few relevant precedents. While other uranium-containing superconductors, such as URhGe^26^ and UCoGe^1^, exhibit field stabilized reentrant superconductivity at specific angles, these phases occur in proximity to ferromagnetic quantum critical points, whereas UTe_2_ does not magnetically order below 1.4 GPa^22^. Other proposed explanations for the intense field enhancement of SC_FP_ include lowered dimensionality^20,21^, which can suppress the orbital limiting effects of magnetic fields, or internal exchange fields that counteract the applied external field^3,5,27,28^, leading the conduction electrons to experience smaller total magnetic fields than those applied. The commonality between these hypotheses is the assumption that high-field superconductivity represents an extension of a lower-field superconducting phase. The debate regarding SC_FP_ thus centers upon which established mechanism fortifies low field superconductivity against the deleterious effects of extreme magnetic fields. The assumptions upon which these models are based are incompatible with a superconducting phase which emerges only at extremely high-fields, and such an observation would therefore require a new form of high-field superconductivity to explain.

In this work, we present the first evidence of “orphaned” high-field superconductivity (oSC_FP_) without an accompanying low-field “parent” phase. This unusual configuration has been achieved in UTe_2_ through the controlled introduction of disorder, which destabilizes SC_1_ and SC_2_, while SC_FP_ unexpectedly survives at high-fields. In addition to presenting the first example of exclusively high-field-stabilized superconductivity in a uranium-based system, these findings dramatically limit possible explanations for the stability of high-field superconductivity in UTe_2_ and its relation to lower field superconductivity, demanding a new theoretical framework.

## Results and discussion

In the Orphan UTe_2_ samples studied here, there is no evidence of SC_1_ or SC_2_ in any field orientation in the *bc* plane when the applied field is smaller than 35 T. Instead, the samples are paramagnetic metals which, like their low-field superconducting cousins, show evidence of Kondo lattice effects upon cooling from room temperature. Zero-field resistance measurements demonstrate Fermi-liquid T^2^ dependence below 10 K (See Supplementary Information, Fig. S2) without evidence of a superconducting transition into the SC_1_ phase down to 110 mK, well below its expected critical temperature, which usually ranges from 1.6 K to 2.1 K^2,3,5,9,29–32^. Disorder scattering, and thus approximate crystalline quality, is roughly estimated in metallic samples by dividing the resistivity at room temperature by the resistivity at 0 K (residual resistivity ratio: RRR = $\frac{{R}_{300K}(\varOmega )}{{R}_{0K}(\varOmega )}$), estimated in our case by extrapolating the T^2^ fit 0-field data. Samples with a clear SC_1_ transition show a great deal of variation in this regard, and can range from the typical^2^ RRR = 18–40 all the way to a reported value of 1000 for exceptionally clean samples^29^. Little progress^27^ has been made towards evaluating the relative sensitivities to disorder of the various superconducting phases, especially at high-fields.

UTe_2_ crystals with no SC_1_ transition usually have a RRR ≲ 5, which implies a high degree of disorder^33,34^. While the value reported herein for Orphan UTe_2_, RRR ≈ 7, is slightly out of this range, it still indicates that these samples are likewise quite disordered. To better understand the relative fragility of the low and high-field superconducting phases, we compare the extraordinarily robust oSC_FP_ phase diagram of Orphan UTe_2_ with two additional crystals. For both additional crystals, ${{T}_{c}}_{{{SC}}_{1}}$ ≈ 1.85 K, which indicates very good quality. However, the large variation of RRR values for “Low-R” (RRR = 8) and “High-R” (RRR = 64), crystals of low-field superconducting UTe_2_ is atypical for any two superconductors with the same chemical formula and T_c_ value. This intimates that the scattering mechanisms that determine RRR in these samples are not identical to the determinants of T_c_.

**Fig. 1:**
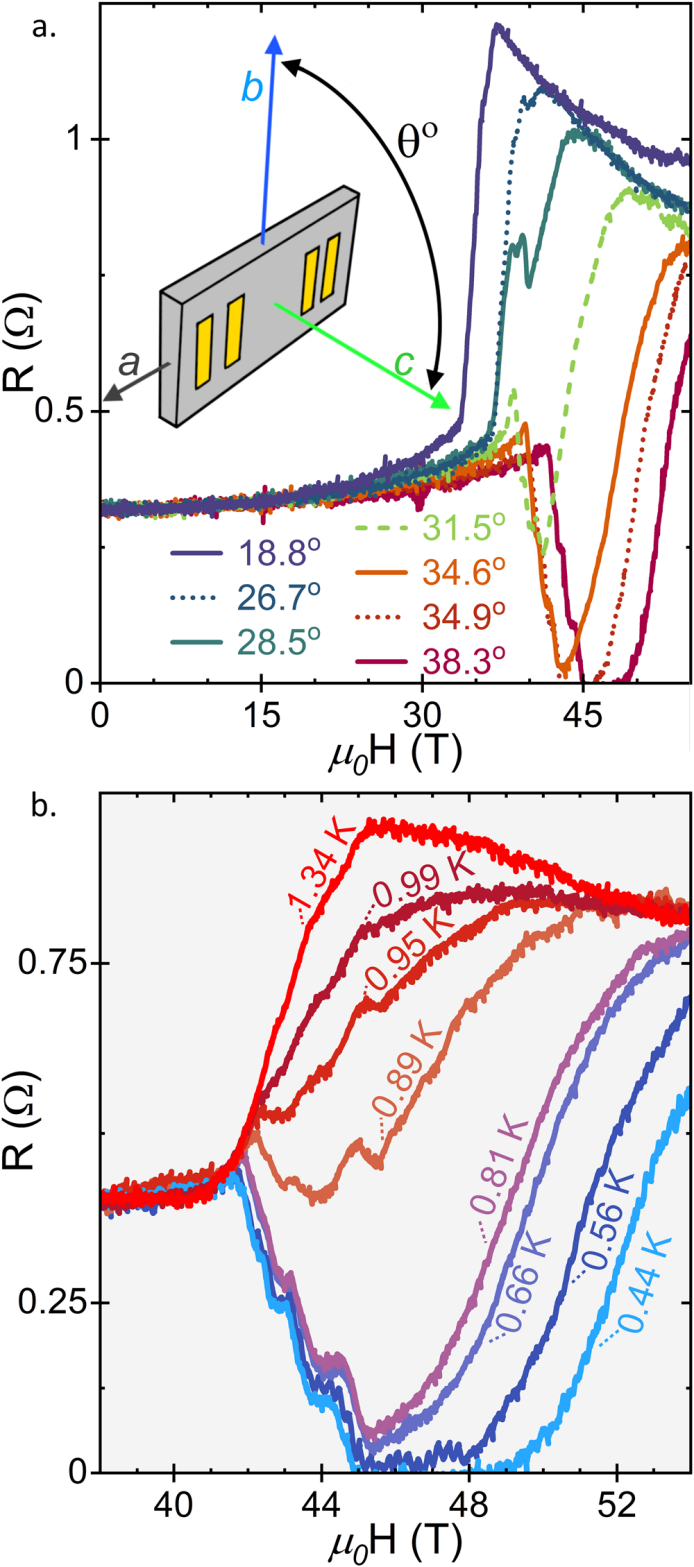
Magnetoresistance of orphan superconducting UTe_2_ at select angles and base temperature, or at fixed angle and select temperatures. **a** Base temperature (~0.5 K) magnetoresistance (0 T < H < 55 T) of orphan superconducting UTe_2_ at base temperature at select angles near the oSC_FP_ phase transition (angles are in degrees from crystallographic *b* to *c*). The large jumps in resistance near 35 T indicate the metamagnetic transition, *H*_*m*_. Inset is an artistic render of the four-wire experimental setup with wires attached to four gold pads on the (001) sample face. **b** Magnetoresistance of orphan superconducting UTe_2_ at with field applied at θ = 38.3^o^ from *b* to *c*, measured at several temperatures from 1.34 K to base.

**Fig. 2:**
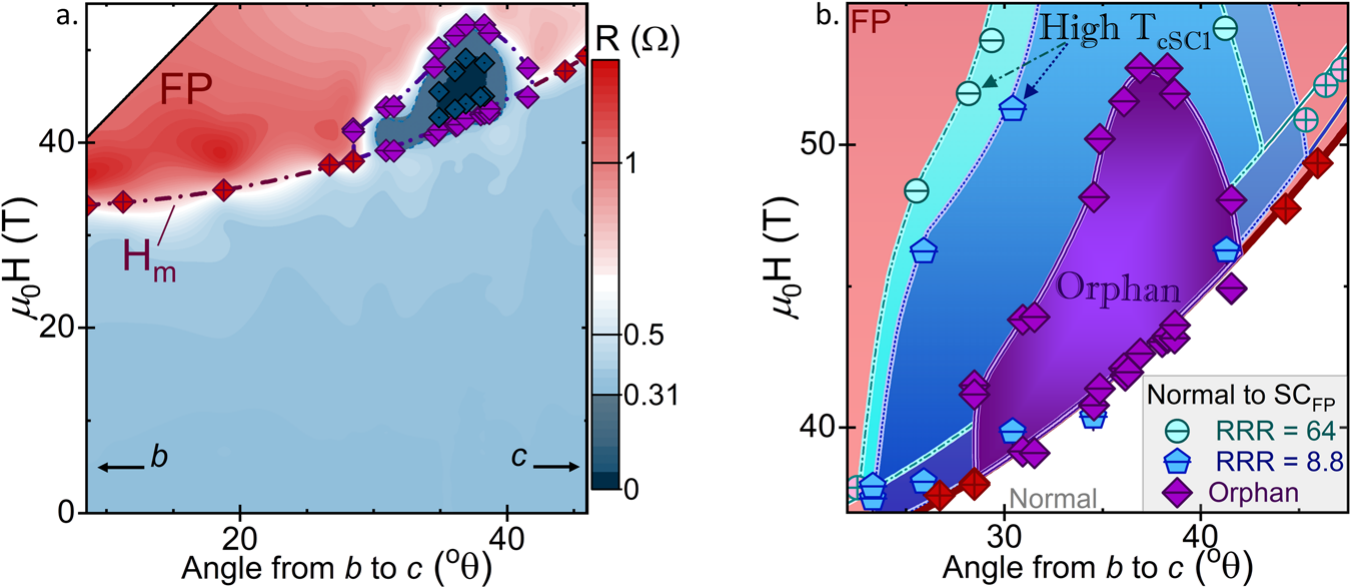
Field-Angle Phase Diagrams at Base Temperature. **a** Phase diagram of oSC_FP_ at base temperature (approx. 0.5 K), with color indicating total resistance. Circled dark blue regions between 30 and 44^o^ are where the sample resistance falls below the average low field normal state value (~0.31 Ω) and the darkest color, bounded by dot-center diamonds, indicates zero resistance. Superconducting transitions and transitions from the low field normal to field polarized normal states (defined by 50% of the transition) are indicated by purple “-” crossed and red “+” crossed diamonds, respectively. **b** Comparison of the oSC_FP_ (purple “-” crossed) to SC_FP_ in ${{T}_{c}}_{{{SC}}_{1}}\approx 1.85{K}$ RRR = 64 (- crossed circles) and RRR = 8.8 (- crossed pentagons) crystals between *H* = 35–55 T and θ = 20–50^o^. In all cases, the normal state (below H_m_) is indicated in white, and the FP normal state (above Orphan UTe_2_ *H*_*m*_) in red. Best fit lines for each *H*_*m*_ and H_c2_ (dash-dot, short-dot, and solid for RRR = 64, 8.8, and Orphan UTe_2_ crystals, respectively) are intended as guides to the eye.

As shown in Fig. 1 and Fig. 2, the metamagnetic transition, *H*_*m*_, occurs just below 35 T along the *b* axis in the Orphan UTe_2_. This value is slightly lower than previous observations of *H*_*m*_ reported from low-field-superconducting samples of UTe_2_^3,5,18,35–37^, and lower than the metamagnetic transitions recorded for both Low-R and High-R UTe_2_ (Fig. 2b). Nevertheless, the field value of this transition still corresponds to the temperature value of a maximum in the magnetic susceptibility with field along *b*, *T*_*χ*_^*max*^ ≈ 35 K, previously reported for both nonsuperconducting^38^ and superconducting^4^ UTe_2_. A similar feature is known in heavy fermion paramagnets with metamagnetic transitions, implying in those cases that H_m_ and T_χ_^max^ are related by a single energy scale^39^. The agreement between the energy scales associated with *T*_*χ*_^*max*^ and H_m_ is also important in UTe_2_^13,35,36^ and reflects the Kondo hybridization energy scale, as further observed in scanning tunneling microscopy^13^ and magnetic excitations in inelastic neutron scattering experiments^40^. These results show that the heavy fermion state in UTe_2_ is a robust characteristic.

We now consider the field-induced orphan superconducting phase that occurs at fields greater than *H*_*m*_ in the field polarized state. This oSC_FP_ phase, with boundaries defined here as 50% of the observed transition, emerges close to a 29^o^ offset from *b* to *c* and extends to 42^o^ (Fig. 1a). The narrower angular range of the oSC_FP_ is striking when compared to typical SC_FP,_ which extends from 25^o^ to 42^o^ in crystals with higher RRR (Fig. 2b, see Fig. S4 in Supplementary Information for comparison with published data^3,35,36,41^). Likewise, the field range oSC_FP_ is reduced, with an upper bound of 52 T. Previous reports have extrapolated the maximum field of SC_FP_ to above 65 T in samples with ${{T}_{c}}_{{{SC}}_{1}}\approx 1.6$ K^3,5,25^. Nevertheless, in terms of magnetoresistance (Fig. 1), the transitions into the FP and SC_FP_ states are qualitatively similar to those in other samples. Note two important features: relatively wide transitions as a function of field and a limited range of zero resistance, both as measured at 0.5 K. The zero-resistance state is centered at 36^o^, which is far from the crystallographic (0 1 1) direction, situated at 23.7 ^o^, suggesting that there is not a direct relationship between the two, which has been previously hypothesized^4^.

**Fig. 3:**
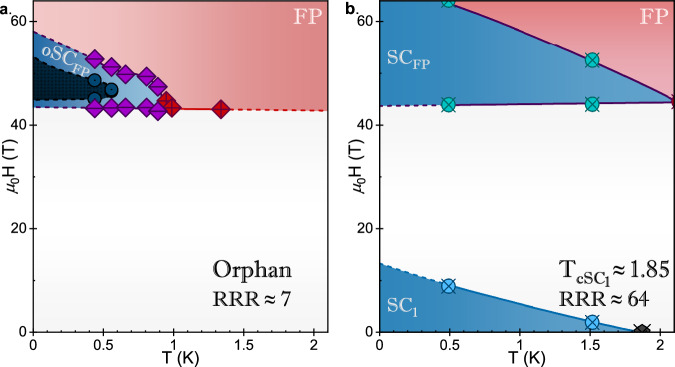
Comparison of Field-Temperature Phase Diagrams for Orphan Superconducting and High ${{T}_{c}}_{{{SC}}_{1}}$, High RRR UTe_2_. In both cases, the field polarized normal state (FP) is indicated in red, and regions of superconductivity (SC_1_ at low field and SC_FP_ at high field) are in dark blue and bounded by lines (solid in between temperature points, dashed when extrapolating) intended as guides to the eye. See **Supplemental Information** for detailed information regarding the determination of phase boundaries and angles. **a** The field-temperature phase diagram of orphan high field superconductivity in Orphan UTe_2_ at 38.3^o^ offset between *b* and *c*. Points between which *R* = 0 Ω are designated with dark blue dotted circles, and the regions between temperature points have been interpolated a dashed-blue boarder and estimated to *T* = 0 K. Dashed lines between FP and either the low field normal state or SC_FP_ indicate regions where this boarder has been estimated to match scaling with **b**. Purple “-” crossed and red “+” crossed diamonds indicate 50% of resistive transitions between phases. **b** The field-temperature phase diagram of SC_FP_ in ${{T}_{c}}_{{{SC}}_{1}}\approx 1.85K$, High-R crystal. Green and blue crossed circles indicate transitions to SC_1_ and SC_FP_, respectively as indicated by changes in PDO frequency (see **SI** for more detail). The 0 applied field ${{T}_{c}}_{{{SC}}_{1}}$, indicated by a black crossed pentagon, was determined via four wire resistance in a Quantum Design Physical Properties Measurement System.

The temperature dependence of oSC_FP_ gives further information about the unprecedented robustness of the superconductivity at these high-fields. The zero resistance state persists to just above 0.5 K (Fig. 3a), and a superconducting envelope persists to almost 0.9 K. All resistive signatures of superconductivity are suppressed by 1 K. This temperature differs dramatically from the value of 1.5 K reported before in samples exhibiting low field superconductivity^3^, and even more so from the high ${{T}_{c}}_{{{SC}}_{1}}$ High-R sample (Fig. 3b). As shown in Fig. 3b, the critical temperature of a more-typical SC_FP_ phase is only slightly higher than that of the low field SC_1_ phase. Previously, the similar *T*_*c*_’s reported for SC_1_ and SC_FP_ led to the inference that the two phases must involve similar pairing energies^3^, or even that SC_FP_ represents true reentrance of SC_1_^42^. These observations led to the expectation that crystallographic disorder should affect T_c_ of both low-field and high-field superconductivity similarly. The observation of oSC_FP_ is at odds with this expectation, further suggesting that the scattering mechanism that dictates the values of RRR is not directly analogous to the strength of the superconducting pair-breaking that sets T_c_.

**Fig. 4:**
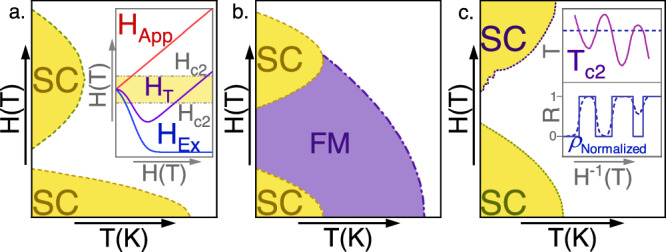
Magnetic field–temperature schematic phase diagrams for superconductivity stabilized by different possible mechanisms. **a** The Jaccarino-Peter compensation effect. An internal exchange field (H_Ex_, blue) opposes the applied field (H_App_) resulting in reentrant superconductivity when the total internal field (H_T_, purple) is smaller than H_c2_. **b** Stabilization of ferromagnetic superconductivity near a quantum critical point. Strong magnetic fluctuations due to the destabilization of long-range magnetism enhance the superconducting pairing. Superconductivity can survive at and on either side of the QCP. **c** Superconductivity stabilized near the quantum limit. The upper critical field of reentrant superconductivity in this case is oscillatory in inverse field.

Relevant theoretical attempts to describe high field superconductivity generally require the presence of zero-field superconductivity^2,3,5,19–21,43^, an assumption which has been reinforced by experimental evidence that high-field superconductivity is typically affected more strongly by temperature and disorder than low field superconductivity^26,44^. It is therefore surprising to see the presumptive fragile phase without its presupposedly more robust neighbor in Orphan UTe_2_, and it will be instructive to review these mechanisms in light of the recontextualization demanded by the orphan SC_FP_ phase. The magnetic field dependence of the superconductivity due to these theoretical mechanisms is illustrated in Fig. 4.

Recently, the Jaccarino-Peter mechanism has been proposed as a likely candidate for the stabilization of SC_FP_ in UTe_2_^25^. This mechanism is believed to be relevant to reentrant superconductivity in organic superconductors and several chevrel phases^45–47^. It involves an internal exchange field generated by the short-range magnetic fluctuations of localized moments, which opposes the applied magnetic field and reduces the total field^48^, allowing superconductivity to persist to higher external fields than it otherwise should (Fig. 4). This exchange field can lead to reetrance, as in the Chevrel phase Eu_0.75_Sn_0.25_Mo_6_S_7.2_Se_0.8_, in which zero-field superconductivity appears below 3.9 K and is suppressed by 1 T^45^. Above 4 T, the external field begins to adequately compensate for the internal exchange field, and superconductivity returns, persisting to approximately 22 T^45^. A similar mechanism is argued to be relevant to field-stabilized superconductivity in the antiferromagnetic insulator λ-(BETS)_2_FeCl_4_. Chemical substitution experiments show that the high-field range of the superconductivity is decreased when antiferromagnetism is destabilized and have been interpreted to indicate that λ-(BETS)_2_FeCl_4_ may have a “hidden” superconducting phase that competes with the antiferromagnetic internal field^49^.

It was pointed out previously that the Jaccarino-Peter mechanism is likely not appropriate for UTe_2_^3^ because this effect requires localized moments and is typically observed in experiment over a narrow angular field range^48^. This contrasts sharply with the weak paramagnetic response of UTe_2_, the substantial angular range of SC_FP_, and the very large magnetic field scale. This inconsistency is reinforced by the new observations of Orphan SC_FP_. The absence of zero field superconductivity without magnetic order to generate a negative exchange field at *H* > 0 almost entirely precludes the compensation-effect as the primary field-stabilizing mode in UTe_2_

Another proposed explanation is that SC_FP_ is stabilized by ferromagnetic fluctuations^2^, similar to field-reinforced superconductivity observed in ferromagnetic superconductors UCoGe^50^ and URhGe^51^ (Fig. 4). In this model, stabilizing longitudinal spin fluctuations arise near a second-order ferromagnetic transition driven by magnetic field^52^. Low field magnetometry measurements at ambient^38^ and high pressure^53^ imply that UTe_2_ lies similarly at the cusp of magnetic order. However, UTe_2_ strongly differs from the superconductors described by the spin-fluctuation model; these materials exhibit both long range magnetic order and low-field superconductivity which precede a field-reentrant superconducting phase^50,51^ For example, spin fluctuations near a metamagnetic spin reorientation lead to reentrant superconductivity in URhGe, and strongly enhance ${{T}_{c}}_{{RE}}$ over the *H* = 0 critical temperature. The low field and reentrant superconducting transition temperatures in URhGe are highly sensitive to sample quality^26,54^. However, when the initial T_c_ boost from enhanced magnetic fluctuations near the metamagnetic field is accounted for, the ordering temperatures of the two phases are almost equally affected by disorder. In fact, the reentrant phase appears to be the slightly more fragile of the two^26^.

Another mechanism for stabilizing high field superconductivity involves field-induced Landau level broadening near the quantum limit^43^. Mean field theory predicts that in applied fields strong enough to constrain electrons to the lowest Landau levels, T_c_ will increase in an oscillatory manner as a function of applied field, reflecting an enhancement of superconducting stability due to the Landau-level structure^43^ (Fig. 4). It has even been hypothesized that approaching the extreme quantum limit could suppress the negative effects of disorder on T_c_ in the high-field regime^43^. Typically the field strength required for this is far beyond the Pauli limit for spin-singlet superconductors^43,55^. Landau-level stabilized superconductivity is therefore most likely to be realized in spin-triplet superconductors. Indeed, high pressure measurements of resistance in low-field-superconducting UTe_2_ show possible precursor effects quantized with the signature 1/H relation to SC_1_ and SC_FP_^22^. However, this model is not without controversy: it has been argued that “unless the [Landé] g-factor is exactly 0^56^,” which is not true in UTe_2_^27^, “re-entrant superconductivity can be expected only if there is a superconducting transition in zero field^56^.” Moreover, a low-dimensional electronic structure is usually assumed for models of superconductivity near the quantum limit^43^, and such a structure could not be inferred in UTe_2_ from angle-resolved photoemission spectroscopy^57^. Recent de Haas van Alphen oscillation measurements of low-field superconducting UTe_2_ suggest quasi-two-dimensional cylindrical electron and hole Fermi surface sections^58^. However, the Fermi surface has three-dimensional characteristics^59–61^, and the inverse-field periodicity implies a small orbit that has yet to be conclusively demonstrated. A separate theoretical analysis has proposed that SC_FP_ in UTe_2_ may be stabilized near the quantum limit by a Hofstadter butterfly regime of Landau level quantization with large superlattices^62^. This stabilization regime would, if accurate, indicate the existence of an even higher field phase beyond SC_FP_, located at approximately 90 T^22,62^, and moreover that the quantum limit field has somehow been lowered from the H > 100 T region inferred from recently reported^59,60^ quantum oscillation frequencies. Furthermore, confirmation of this model would ideally involve observation of superconductivity in multiple Landau levels, requiring challenging measurements performed at significantly higher magnetic fields.

The above inconsistencies show that SC_FP_ is likely not a field-stabilized version of SC_1_ and its pairing state should be considered separately. In other words, SC_FP_ and SC_1_ are substantially different superconducting phases, could involve different superconducting pairing mechanisms, and their gap structure and size are different. The lack of a parent superconducting instability makes it more remarkable that SC_FP_ is stable at such high magnetic fields, as the dominant theoretical descriptions of high-field superconductivity presuppose a low-field antecedent. While none of the three scenarios we have discussed anticipate oSC_FP_, other potential explanations such as the invocation of “hidden” superconductivity in UTe_2_, similar to that in the Chevrel^47^ case, would require even more a priori assumptions and cannot be considered useful models at this stage. We must conclude that further understanding of SC_FP_ specifically, and field stabilized superconductivity as a whole, demand the further development of models of high-field superconductivity that do not evolve from a low field superconducting phase.

## Methods

All samples were grown as single crystals via chemical vapor transport with iodine oas the transport agent. Orphan UTe_2_ crystals were grown over one week as thin plates approximately 3 mm in length from a 2:3 U:Te ratio in a two zone furnace set to 800 ^o^C and 710 ^o^C in the charge and growth zones, respectively. The Low-R and High-R samples were grown in a two zone furnace at 900 ^o^C (charge zone) and 830 ^o^C (growth zone) over two weeks with starting U:Te ratios of 5:9 and 2:3, respectively. At the end of the growth period, transport was quenched by turning off power to the heating elements. Crystallographic orientation was identified from the crystal habit.

Zero-field resistance measurements to 100 mK were performed on a Quantum Design Physical Property Measurement System (PPMS) using the adiabatic demagnetization refrigerator (ADR) option. For high field measurements, crystals were mounted on a cryogenic single axis goniometer^63^ at the National High Magnetic Field Laboratory (NHMFL), Los Alamos, NM and rotated between the (010) and (001) faces at applied fields of up to 55 T or up to 60 T. Both high field magnetoresistance and proximity diode oscillator measurements were performed using a 65 T short-pulse magnet. Identification of commercial equipment does not imply recommendation or endorsement by NIST.

### Supplementary information

Supplementary Information

Peer Review File

## Data Availability

The phase boundaries represented in Figs. 1–3 are available as tables and their definitions explained in detail in the Supplementary Information. Raw magnetoresistance and PDO data files are publicly available in the OSF Repository: 10.17605/OSF.IO/Q3HSE^64^. All other data are available from the corresponding authors upon request.
