# Supplementary material for: Orphan high field superconductivity in non-superconducting uranium ditelluride
Source: Nat Commun. 2024 Apr 20;15:3378. doi: 10.1038/s41467-024-47090-1 (PMC11032386; doi:10.1038/s41467-024-47090-1)
Supplement: Supplementary file 1 — Supplementary Information [file 41467_2024_47090_MOESM1_ESM.pdf]

Orphan High Field Superconductivity in Non-Superconducting Uranium Ditelluride

Supplementary Information

Corey E. Frank,[1,2*] Sylvia K. Lewin,[1,2] Gicela Saucedo Salas,[2,1] Peter Czajka,[1,2] Ian M. Hayes,[2] Hyeok Yoon,[2] Tristin Metz,[2] Johnpierre Paglione,[2,3] John Singleton,[4] Nicholas P. Butch[1,2*]

1. NIST Center for Neutron Research, National Institute of Standards and Technology, Gaithersburg, MD, USA
2. Maryland Quantum Materials Center, Department of Physics, University of Maryland, College Park, MD, USA
3. Canadian Institute for Advanced Research, Toronto, Ontario M5G 1Z8, Canada
4. National High Magnetic Field Laboratory, Los Alamos National Laboratory, Los Alamos, NM, USA

**To whom correspondence should be addressed.*

Sample Preparation and Resistivity Measurements at 0 T applied Magnetic Field

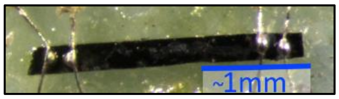

Figure S 1 Approx. 2.5 mm crystal of low-field non superconducting UTe_2 grown as a flat plate in the *ab* plane via chemical vapor transport, as described in main text. Crystal is depicted with typical four-wire configuration as used for resistance measurements.

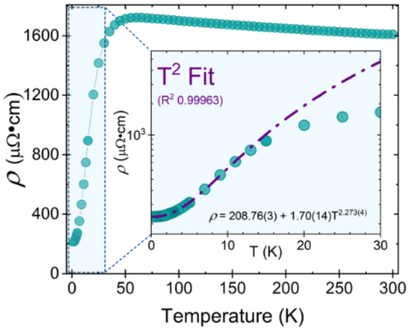

Figure S 2 Preliminary, zero field resistance of the specially prepared low-field non-superconducting UTe_2 sample used for high field magnetoresistance measurements at NHMFL Pulsed Field Facility. At applied field H = 0 T, this sample showed no indication of a superconducting transition to 0.110 K . A T^2 fit of the resistance indicates Fermi-liquid behavior to approximately 10 K, a two order of magnitude range.

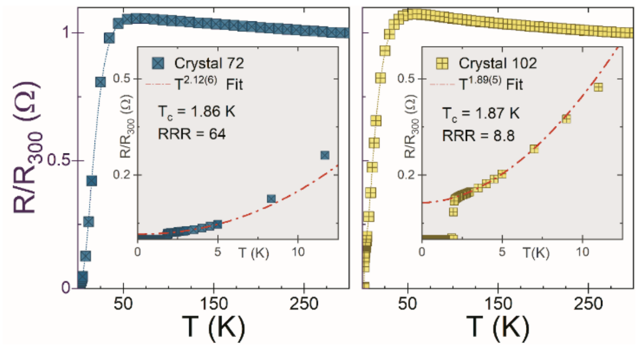

Figure S 3 Preliminary, zero field resistance of high T_c UTe_2 samples used for high field proximity diode oscillator measurements at NHMFL Pulsed Field Facility.

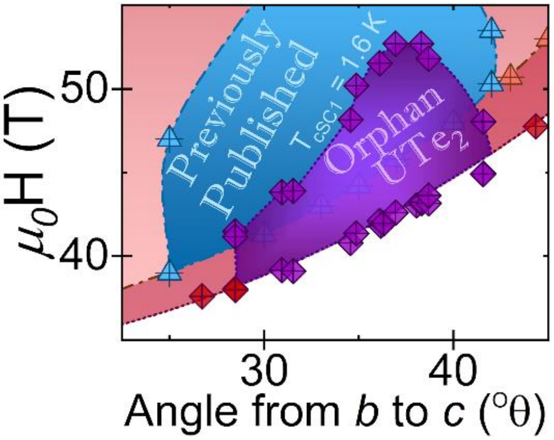

Figure S 4. Phase diagram of resistance in applied field (y-axis) vs angular offset from *b* to *c* for orphan-superconducting UTe_2. Superconducting transitions (defined by 50% of the transition) are shown in purple diamonds, and transitions from the low-field-normal state to the field-polarized-normal state are indicated by red diamonds. The orphan superconducting region is indicated in purple and the field polarized normal state in red, with colors and lines as a guide to the eye. The dashed red line is a fit of H_m in orphan UTe_2, also intended as a guide to the eye. These data are overlayed with previously reported[1] data. Referenced UTe_2 phase boundaries are shown as simple, smooth blue lines for clarity and are intended as a tool of contextualization. Solid blue regions indicate superconductivity in archetypical ($T_{c_{SC1}} \approx 1.6$ K) UTe_2 only, and the solid red region indicates the angular range of the field polarized regime in the *bc* plane.[1]

Data Processing and Definition of Phase Boundaries – Orphan UTe_2

For phase diagrams shown in the main text, transitions were defined as 50% of the difference in resistance between the normal and field polarized, the normal and superconducting, or the superconducting and field polarized states, respectively. To define transitions from the normal state or superconducting states to the field polarized regime, or from the normal state to oSC_{FP}, a point was chosen where resistance was a relative minimum (R_{min}) proximal to the inflection point. For a full superconducting transition, $R_{min} = 0\ \Omega$. The point of maximum resistance, R_{max}, after (before) this transition defines the normal state resistance in the FP (before the oSC_{FP}) phaseOnce the limits were defined, the 10%, 50%, and 90% resistance values between them were calculated, and the corresponding applied field values found on each curve (**Fig S.5**). These values are listed in the tables below. In cases where the maximum value of resistance fell outside of the measured value for the respective field pulse, R_{max} was estimated. This estimation was performed by plotting R_{max} vs angle for well-defined pulses as can be seen in **Fig S.6**. Data were combined with data from Fig 1 in the main text to generate Fig 2 in the main text.

---

1 Ran, S. *et al.* Extreme magnetic field-boosted superconductivity. *Nature Physics* **15**, 1250-1254, doi:10.1038/s41567-019-0670-x (2019).

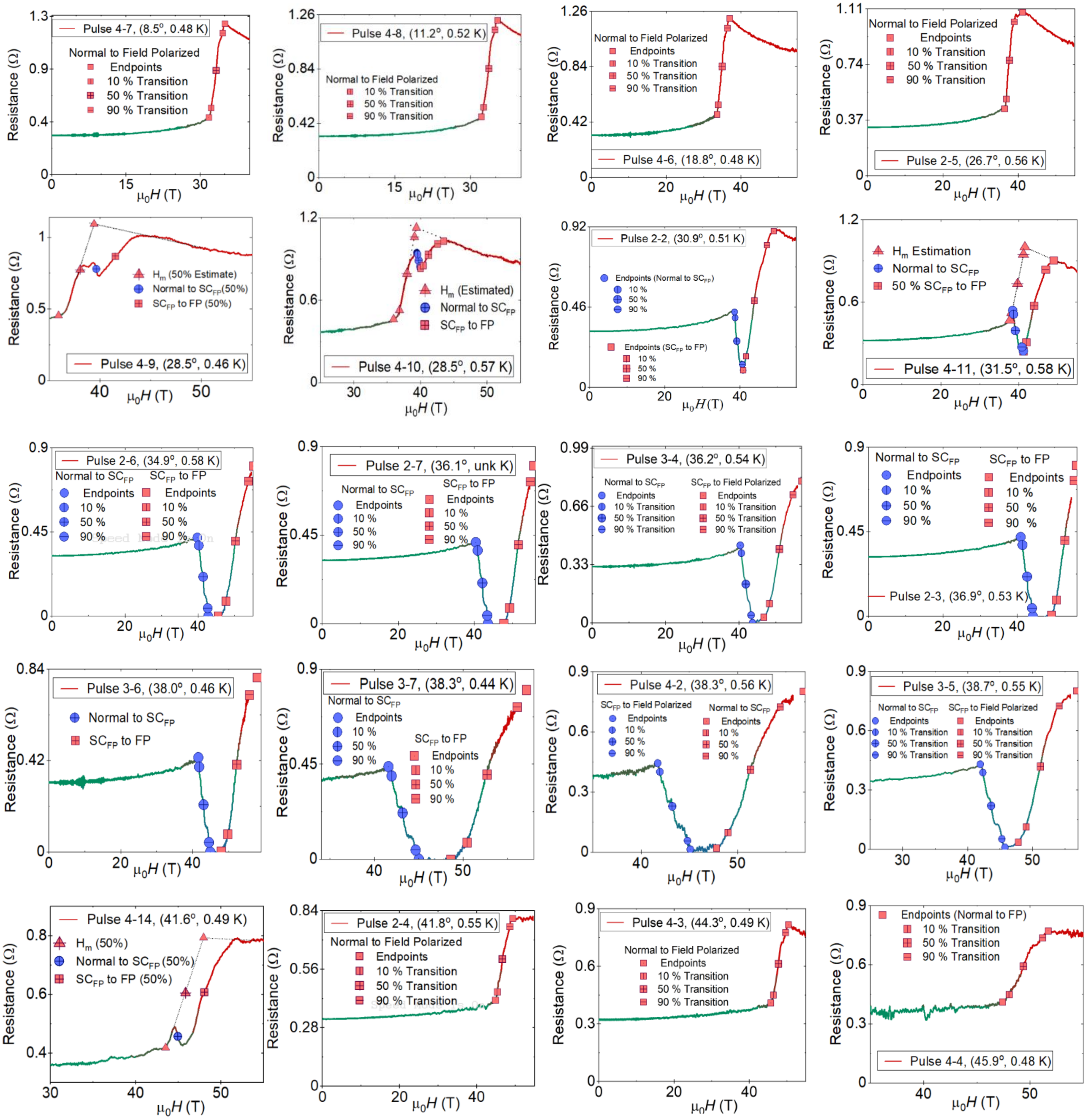

Figure S 5 Base-temperature resistance curves showing a transition to the field polarized state (red squares), from the normal state to oSC_{FP} (blue circles), and in some cases an estimation of H_m when interrupted by a partial superconducting transition (red triangles). The interior of each shape indicates the endpoints (open shapes), 10 % (vertical center line), 50 % (crosses), and 90 % (horizontal crosses) points of the respective transitions. Resistance curves shown here are from the pulsed field magnet downsweep only. Data used to construct Fig. 1 in the main text.

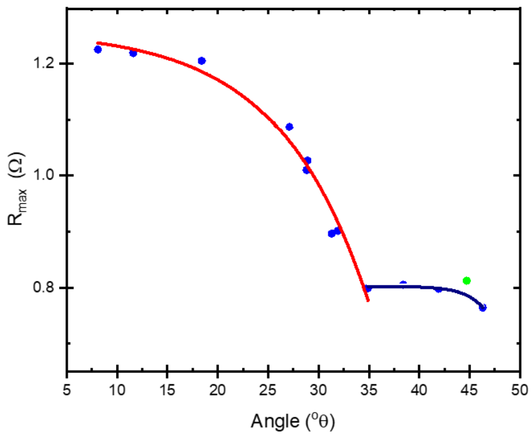

Figure S 6 Maximum resistance (Ohms) of base temperature magnetoresistance measurements vs field angle (degrees from *b* to *c*). Fitting angle vs R_{max} allows for R_{max} to be estimated when R_{max} is outside of measurement range.

Temperature Dependent Measurements on Orphan UTe_2 at θ = 38.3° between *b* and *c*.

Table S. 1 Temperature Dependent H_m (Low Field Normal State to oSC_{FP})

**Temp (K)**	**Angle *θ* (°)**	Starting Values μ₀*H* (T)	*R* (Ω)	End Values μ₀*H* (T)	*R* (Ω)	**50% Transition μ₀*H* (T)**	*R* (Ω)	90% Transition μ₀*H* (T)	*R* (Ω)	10% Transition μ₀*H* (T)	*R* (Ω)
**0.44**	**38.3**	41.6	0.4386	45.0	0	**43.2**	0.2193	44.6	0.0439	41.9	0.3948
**0.56**	**38.3**	41.7	0.4367	45.2	0.0113	**43.3**	0.224	44.8	0.0539	42.0	0.3941
**0.66**	**38.3**	41.8	0.4627	45.5	0.0367	**43.3**	0.2497	45.0	0.0793	42.1	0.4201
**0.81**	**38.3**	41.8	0.4635	45.4	0.0572	**43.3**	0.2603	44.9	0.0978	42.1	0.4229
**0.95**	**38.3**	41.2	0.4394	49.4	0.8384	**44.6**	0.6389	47.9	0.7985	41.9	0.4793

Transitions (as defined above) from the low-field normal state into oSC_{FP} at varying temperatures and constant angle. Data used to construct Fig. 2 in the main text.

Table S. 2 Temperature Dependent H_m or H_{C2} (Low Field Normal or oSC_{FP} to Field Polarized Normal State)

**Temp (K)**	**Angle *θ* (°)**	Starting Values μ₀*H* (T)	*R* (Ω)	End Values μ₀*H* (T)	*R* (Ω)	**50% Transition μ₀*H* (T)**	*R* (Ω)	90% Transition μ₀*H* (T)	*R* (Ω)	10% Transition μ₀*H* (T)	*R* (Ω)
**0.44**	**38.3**	48.6	0	51.8	0.8026	**52.7**	0.4013	57.1	0.7224	50.4	0.0803
**0.56**	**38.3**	48.1	0.0294	54.8	0.7538	**51.2**	0.3916	53.8	0.6814	49.0	0.1019
**0.66**	**38.3**	45.5	0.0367	54.5	0.7816	**49.7**	0.4091	52.3	0.7071	47.3	0.1112
**0.81**	**38.3**	45.4	0.0572	54.7	0.8059	**49.3**	0.4315	52.1	0.731	46.9	0.132
**0.89**	**38.3**	43.9	0.4085	51.2	0.8368	**47.3**	0.6227	50.2	0.794	44.5	0.4513
**0.99**	**38.3**	40.8	0.4144	48.6	0.8525	**43.3**	0.6334	45.6	0.8087	41.6	0.4582
**1.34**	**38.3**	40.8	0.4146	45.5	0.9483	**43.0**	0.6815	44.7	0.8949	41.7	0.468

Transitions (as defined above) from the low-field normal state or oSC_{FP} into the field polarized normal state at varying temperatures and constant angle. Data used to construct Fig. 2 in the main text.

Angle Dependent Measurements on Orphan UTe_2 at Base Temperature

Table S. 3 Angle Dependent H_m (Low Field Normal State to oSC_{FP}) for Orphan UTe_2

**Temp (K)**	**Angle θ (°)**	Starting Values $\mu_0 H$ (T)	Starting Values R (Ω)	End Values $\mu_0 H$ (T)	End Values R (Ω)	**50% Transition $\mu_0 H$ (T)**	50% Transition R (Ω)	90% Transition $\mu_0 H$ (T)	90% Transition R (Ω)	10% Transition $\mu_0 H$ (T)	10% Transition R (Ω)
**0.46**	**28.5**	39.4	0.8224	39.7	0.7372	**39.6**	0.7798	39.7	0.7457	39.5	0.8138
**0.57**	**28.5**	39.4	0.9452	40	0.8347	**39.7**	0.8899	39.9	0.8457	39.6	0.9341
**0.51**	**30.9**	38.6	0.4322	40.9	0.0982	**39.2**	0.2652	40.6	0.1316	38.7	0.3988
**0.58**	**31.5**	38.4	0.5404	41.2	0.2423	**39.1**	0.3914	40.8	0.2721	38.7	0.5106
**0.57**	**34.6**	39.6	0.4758	42.6	0.0376	**40.8**	0.2567	42.3	0.0814	39.8	0.432
**0.58**	**34.9**	39.8	0.4195	42.7	0	**41.4**	0.2097	42.6	0.0419	40.2	0.3775
**unk**	**36.1**	40.4	0.4131	43.6	0	**42.1**	0.2066	43.4	0.0413	40.9	0.3718
**0.54**	**36.2**	40.3	0.439	43.7	0	**41.8**	0.2195	43.3	0.0439	40.6	0.3951
**0.59**	**36.3**	40.5	0.4573	43.8	0.0222	**42**	0.2398	43.4	0.0657	40.8	0.4138
**0.53**	**36.9**	40.9	0.4214	44.2	0	**42.6**	0.2107	44	0.0421	41.4	0.3793
**0.46**	**38**	41.6	0.4338	44.9	0	**43**	0.2169	44.5	0.0434	41.8	0.3904
**0.44**	**38.3**	41.6	0.4386	45	0	**43.2**	0.2193	44.6	0.0439	41.9	0.3948
**0.56**	**38.3**	41.7	0.4437	45.1	0.0144	**43.2**	0.2291	44.8	0.0573	42	0.4008
**0.48**	**38.7**	41.8	0.4957	45.2	0.0746	**43.2**	0.2852	44.9	0.1167	42	0.4536
**0.55**	**38.7**	42	0.4304	45.7	0.0099	**43.6**	0.2201	45.3	0.0519	42.4	0.3883
**0.49**	**41.6**	44.6	0.4887	45.5	0.4263	**44.9**	0.4575	45.3	0.4326	44.7	0.4825

Transitions (as defined above) from the low-field normal state into oSC_{FP} at varying angles from *b* to *c* and base temperature (approximately 500 mK). Data used to construct Fig. 1 in the main text.

Table S. 4 Angle Dependent H_{C2} (oSC_{FP} to Field Polarized Normal State) for Orphan UTe_2*

**Temp (K)**	**Angle θ (°)**	Starting Values $\mu_0 H$ (T)	Starting Values R (Ω)	End Values $\mu_0 H$ (T)	End Values R (Ω)	**50% Transition $\mu_0 H$ (T)**	50% Transition R (Ω)	90% Transition $\mu_0 H$ (T)	90% Transition R (Ω)	10% Transition $\mu_0 H$ (T)	10% Transition R (Ω)
**0.46**	**28.5**	39.9	0.7293	43.8	1.008	**41.5**	0.8686	42.9	0.9801	40.2	0.7572
**0.57**	**28.5**	40	0.8347	43.5	1.0312	**41.2**	0.933	42.6	1.0116	40.4	0.8543
**0.51**	**30.9**	40.9	0.0982	49	0.8946	**43.8**	0.4964	47.2	0.815	41.6	0.1778
**0.58**	**31.5**	41.2	0.2423	49	0.904	**43.9**	0.5732	46.9	0.8379	41.9	0.3085
**0.57**	**34.6**	43.2	0.0319	53.9	0.7945	**48.2**	0.4132	51.7	0.7183	44.8	0.1082
**0.58**	**34.9**	45.5	0	Estimated	0.8031	**50.2**	0.4016	53.8	0.7228	47.6	0.0803
**unk**	**36.1**	47.7	0	Estimated	0.803	**51.5**	0.4015	54.7	0.7227	49.2	0.0803
**0.54**	**36.2**	46.7	0.032	Estimated	0.803	**51**	0.4175	54.7	0.7259	48.3	0.1091
**0.59**	**36.3**	45.9	0.0335	Estimated	0.803	**50.3**	0.4182	53.8	0.7261	47.5	0.1104
**0.53**	**36.9**	49.1	0.007	Estimated	0.8029	**52.7**	0.405	55.2	0.7233	50.4	0.0866
**0.46**	**38**	47.9	0.0025	Estimated	0.8027	**52.3**	0.4026	55.7	0.7227	49.7	0.0825
**0.44**	**38.3**	48.6	0	Estimated	0.8026	**52.7**	0.4013	56.1	0.7224	50.4	0.0803
**0.56**	**38.3**	47.8	0.0213	Estimated	0.8026	**51.3**	0.4119	54.4	0.7245	49	0.0994
**0.48**	**38.7**	48.7	0.1146	Estimated	0.8731	**51.8**	0.4938	54.5	0.7972	49.7	0.1904
**0.55**	**38.7**	47.8	0.0375	Estimated	0.8025	**51.2**	0.42	54	0.726	49	0.114
**0.49**	**41.6**	43.5	0.4186	51.8	0.7902	**48**	0.6083	50.6	0.7539	46.6	0.4627

Transitions (as defined above) from SC_{FP} into the field polarized normal state at varying angles from *b* to *c* and base temperature (approximately 500 mK). Data used to construct Fig. 1 in the main text. Estimation of H_{max} from utilized where "fitting function" is listed in the table.

Table S. 5 Angle Dependent H_m (Low Field to Field Polarized Normal State) for Orphan UTe_2

**Temp (K)**	**Angle θ (°)**	Starting Values $\mu_0 H$ (T)	Starting Values R (Ω)	End Values $\mu_0 H$ (T)	End Values R (Ω)	**50% Transition $\mu_0 H$ (T)**	50% Transition R (Ω)	90% Transition $\mu_0 H$ (T)	90% Transition R (Ω)	10% Transition $\mu_0 H$ (T)	10% Transition R (Ω)
**0.48**	**8.5**	31.7	0.4699	35	1.2319	**33.2**	0.8509	34.5	1.1557	32.2	0.5461
**0.52**	**11.2**	32.1	0.4711	35.4	1.219	**33.6**	0.8451	34.9	1.1442	32.6	0.5459
**0.48**	**18.8**	33.5	0.4802	36.9	1.2064	**34.9**	0.8433	36.3	1.1337	33.9	0.5528
**0.56**	**26.7**	36.3	0.4473	41.1	1.0893	**37.6**	0.7683	38.9	1.0251	36.8	0.5115
**0.46**	**28.5**	35.9	0.4545	39.4	1.0932	**38**	0.7739	39.1	1.0294	36.8	0.5184
**0.57**	**28.5**	36	0.4613	39.4	1.1282	**38**	0.7948	39.1	1.0615	36.9	0.528
**0.58**	**41.6**	43.3	0.4118	47.1	0.8263	**45.5**	0.619	46.8	0.7848	44.1	0.4532
**0.55**	**41.8**	44.8	0.4132	49.2	0.8043	**46.6**	0.6087	48.5	0.7652	45.3	0.4523
**0.49**	**44.3**	45.7	0.4089	50.4	0.8163	**47.7**	0.6126	49.6	0.7756	46.4	0.4497
**0.48**	**45.9**	47.4	0.4131	51.7	0.773	**49.4**	0.5931	51.2	0.737	48	0.4491

Transitions (as defined above) from the low field normal state into the field polarized normal state at varying angles from *b* to *c* and base temperature (approximately 500 mK). Data used to construct Fig. 1 in the main text.

Data Processing and Definition of Phase Boundaries – Low-field-superconducting UTe_2

For low-field superconducting crystals, upper critical fields for SC_1 and SC_{FP} were defined from proximity diode oscillator (PDO) measurements as shown in S.7. The metamagnetic transition was defined as the point of maximum slope change, and found by evaluation of the first derivative of the frequency vs applied field curves.

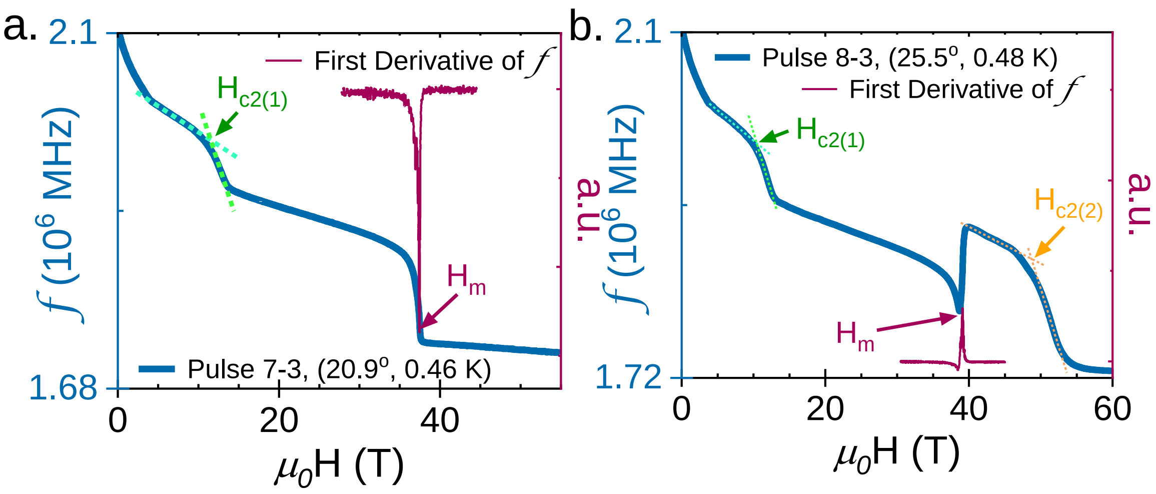

Figure S 7 Example of phase boundary identification showing transitions from SC_1 to the low field normal state and from the low field normal state to (a.) the field polarized normal phase or (b.) SC_{FP} in RRR = 64 samples determined from proximity diode oscillator experiments on field superconducting UTe_2 at base temperature (approximately 500 mK).

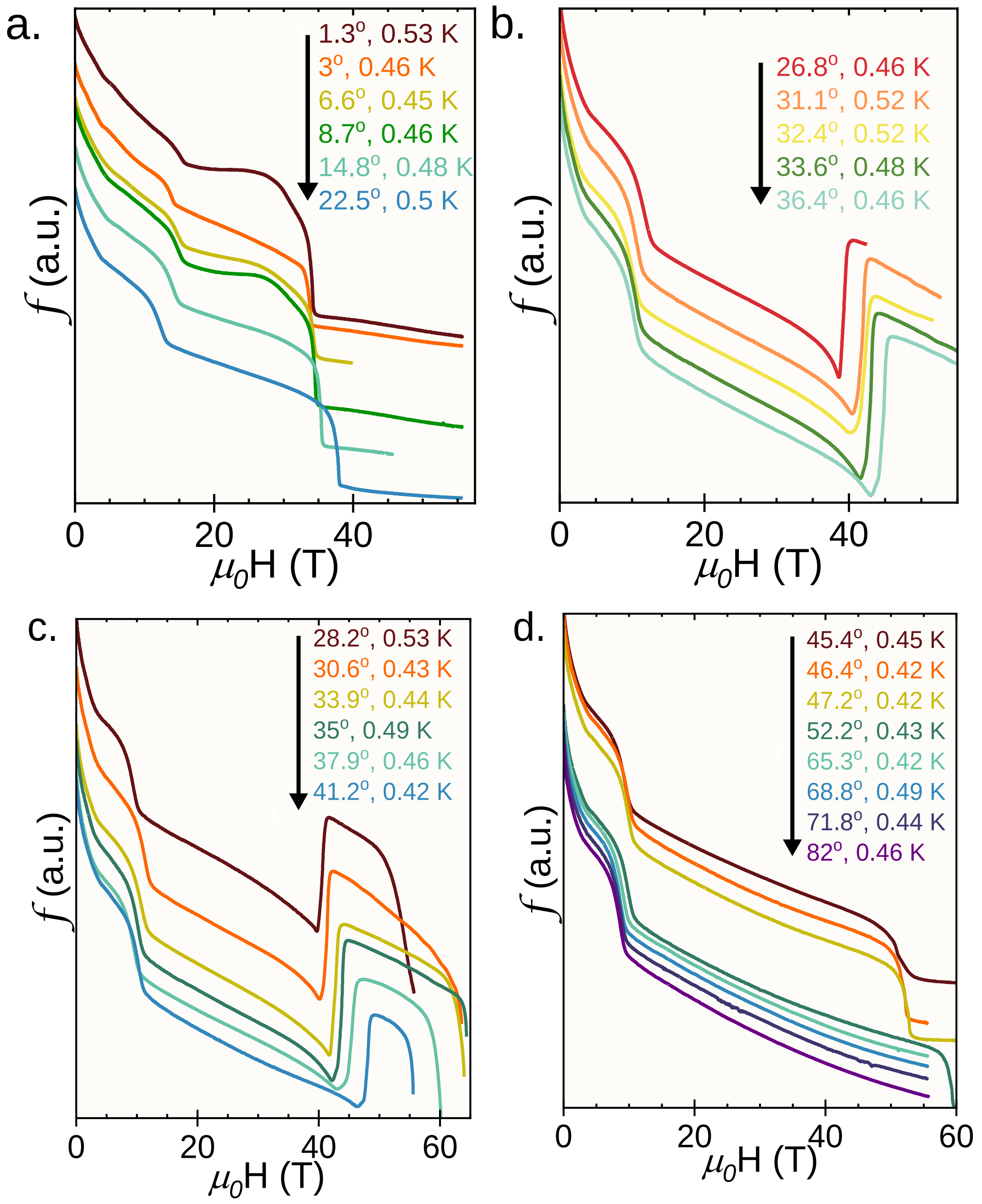

Figure S 8 Base-temperature PDO curves for RRR = 64 low-field-superconducting UTe_2 measured at field orientations offset from crystallographic *b* to *c* by 1.3° – 22.5° (a.), 26.8 ° – 36.4° (b.), 28.2 ° – 41.2° (c.), or 45.4° – 82° (d.). In all cases, vertical units are arbitrary, as individual curves are offset in the in the y axis for visual clarity. Data used to prepare Figure 2 in the main text.

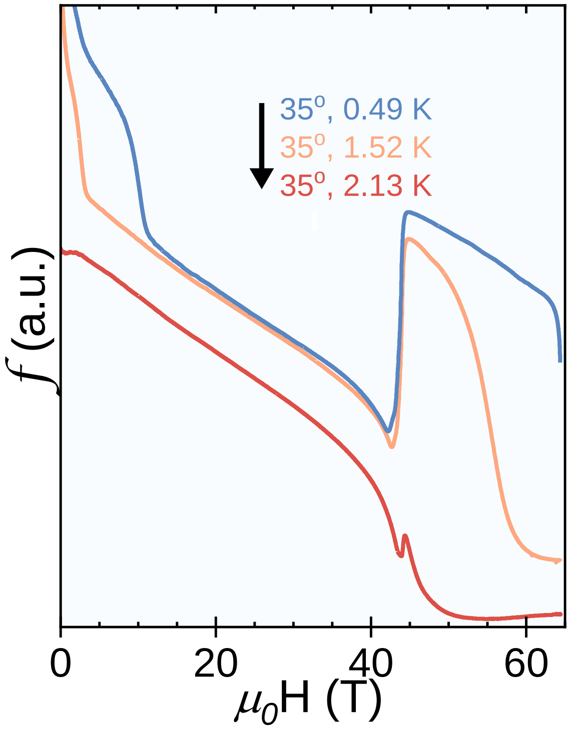

Figure S 9 Temperature dependent PDO curves at a fixed angle, θ = 35° from *b* to *c*, as measured for RRR = 64 low-field-superconducting UTe_2. Vertical units are arbitrary, as individual curves are offset in the in the y axis for visual clarity. Data used to prepare Figure 3 in the main text.

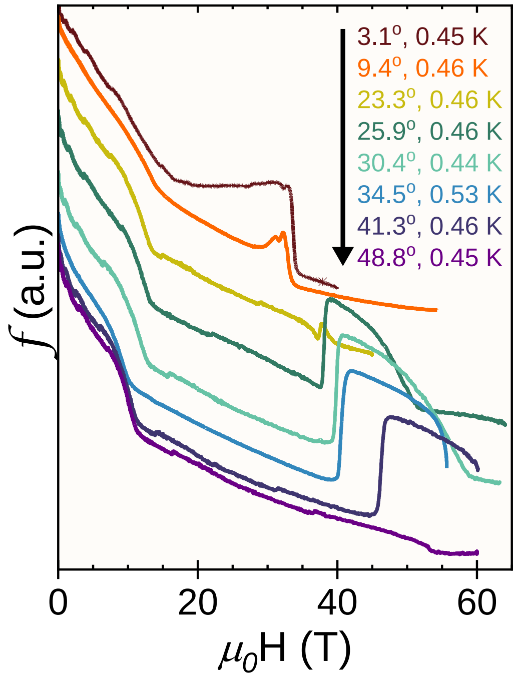

Figure S 10 Angle dependent PDO curves at low temperature as measured for “low” quality (RRR = 8) low-field-superconducting UTe_2. Vertical units are arbitrary, as individual curves are offset in the in the y axis for visual clarity. Data used to prepare Figure 2 in the main text.

Table S. 6 Summary of Phase Transitions from PDO Measurements of RRR = 64 UTe_2

Temp (K)	Angle θ (°)	$H_{c2}(SC_1)$ $\mu_0 H$ (T)	H_m (to FP) $\mu_0 H$ (T)	H_m (to SC_{FP}) $\mu_0 H$ (T)	H_{c2} (SC_{FP}) R (Ω)
0.53	1.3	13.8	34.1		
0.46	3	13.0	33.8		
0.45	6.6	13.6	34.3		
0.46	8.7	13.7	34.6		
0.48	14.8	12.8	35.4		
0.46	20.9	11.3	37.4		
0.5	22.5	10.8	37.9		
0.52	23.9	10.6	37.6		
0.48	25.5	10.3		39.1	48.4
0.46	26.8	10.2		39.3	
0.53	28.2	8.1		40.6	51.8
0.46	29.4	9.5		40.3	53.7
0.43	30.6	10.0		41.3	
0.52	31.3	9.3		42.0	
0.52	32.4	8.6		42.2	
0.48	33.6	9.3		43.1	
0.44	33.9	9.1		42.9	61.3
0.49	34.9	8.9		43.9	64.1
1.52	34.9	2.0		44.0	52.4
2.13	34.9	-		44.5	44.5
0.46	36.4	9.0		44.9	
0.46	37.9	8.1		45.5	58.7
0.42	41.2	8.9		48.1	54.1
0.45	45.4	8.0	50.9		
0.42	46.4	8.7	52.1		
0.42	47.2	8.7	52.7		
0.43	52.2	8.7	59.4		
0.42	65.3	7.7			
0.49	68.8	7.4			
0.44	71.8	7.5			
0.46	82	7.4			

Table S. 7 Summary of Phase Transitions from PDO Measurements of RRR = 8 UTe_2

**Temp (K)**	**Angle θ (°)**	$H_{c2}(SC_1)$ $\mu_0 H$ (T)	H_m (Normal to FP Normal) $\mu_0 H$ (T)	H_m (Normal to SC_{FP}) $\mu_0 H$ (T)	H_{c2} (SC_{FP}) R (Ω)
0.45	3.1	9.2	33.7	--	--
0.46	9.4	10.2	32.8	--	--
0.46	23.3	10.1		37.5	37.9
0.46	25.9	10.5		38.1	46.2
0.44	30.4	9.5		39.8	51.3
0.53	34.5	7.9		40.4	54.8
0.46	41.3	8.4		46.3	59.4
0.45	48.8	8.7	53.2	--	--
