# Supplementary material for: Orphan high field superconductivity in non-superconducting uranium ditelluride
Source: Nat Commun. 2024 Apr 20;15:3378. doi: 10.1038/s41467-024-47090-1 (PMC11032386; doi:10.1038/s41467-024-47090-1)
Supplement: Supplementary file 2 — Peer Review File [file 41467_2024_47090_MOESM2_ESM.pdf]

nature portfolio

Peer Review File

Orphan High Field Superconductivity in Non-Superconducting Uranium Ditelluride

Open Access This file is licensed under a Creative Commons Attribution 4.0 International License, which permits use, sharing, adaptation, distribution and reproduction in any medium or format, as long as you give appropriate credit to the original author(s) and the source, provide a link to the Creative Commons license, and indicate if changes were made. In the cases where the authors are anonymous, such as is the case for the reports of anonymous peer reviewers, author attribution should be to 'Anonymous Referee' followed by a clear attribution to the source work. The images or other third party material in this file are included in the article's Creative Commons license, unless indicated otherwise in a credit line to the material. If material is not included in the article's Creative Commons license and your intended use is not permitted by statutory regulation or exceeds the permitted use, you will need to obtain permission directly from the copyright holder. To view a copy of this license, visit http://creativecommons.org/licenses/by/4.0/.

Editorial Note: This manuscript has been previously reviewed at another journal that is not operating a transparent peer review scheme. This document only contains reviewer comments and rebuttal letters for versions considered at *Nature Communications*.

REVIEWERS' COMMENTS

Reviewer #1 (Remarks to the Author):

This article reports on the occurrence of high-field superconductivity in a sample of UTe2 in which no low-field superconductivity occurs. I have read the new version of the paper and saw that the authors have improved it compared to the previous version.

The methodology used to obtain the experimental results is sound, the experimental findings are convincing, and the data analysis is carefully done. All of this is according to the standards in the field.

I now think that the paper has higher quality than before, but I am not sure it is worth a publication in Nature Communications. The finding to me isn't so surprising and new, and it doesn't directly lead to fundamentally new possible interpretations/insight. The authors claim that only this finding reveals that the high-field phase is of different origin than the low-field phase. However, this is not true. Other scenarios have been considered before (and are discussed and cited in this paper as well) and even the low-field phase diagram consists of two separate phases of possibly different origin. On the other hand I agree with the authors that this is the first finding of this kind and that the complicated phase diagram of UTe2 still lacks out theoretical understanding. But this was the case before as well.

I also have more specific comments/requests of improvement.

Introduction

The introduction is quite lengthy and has a number of repetitions. I suggest the authors to write a sharper version, more to the point.

References

Some of the references should be updated. For example ref 16 which is now published, and 26/27, which are actually one publication with a new title and arxiv version published in Nat. Com.
In the last sentence of the first paragraph in the Results and Discussion section ref. 29 should be cited.

The discussion on RRR lacks giving the RRR of the main sample in this study in the text. This information is only given in fig. 4 a.

The discussion on Quantum-limit induced superconductivity lacks giving numbers of the Quantum limit fields for UTe2 which are simply given by the quantum oscillation frequencies. All of these

frequencies are above 100 T, even for the disputed small 3D Fermi surface. These fields are far above the fields of field-induced SC in UTe_2 and make this scenario unlikely. This should be mentioned.

Figures
Not much care is put into the figures. At some places, things that should be aligned (like axis labels of the same kind in different panels) are not, things that should have the same size, don't have it, etc.
Fig. 2 a and b have way too large labels. It actually reduces readability. Colors of text in the different phases in Fig. 2b are not well chosen (not readable). Point sizes are enormous.
Labels in Fig. 3 are also too big. Same for fig. 4.

Reviewer #2 (Remarks to the Author):

I have carefully read through the revised manuscript and the responses by the authors Corey E. Frank et al. The revised manuscript added new data with RRR=8.8 and updated the phase diagrams for comparison (revised Fig. 2b and Fig. 3b). Below are my comments and responses to the revised manuscript and the authors' rebuttal letter.

First, I'm afraid that the authors didn't carefully read the contents of my report and misunderstood its main points. Below is what I was trying to address in the initial report to Nature Physics: In literature, it has been reported that two superconducting states (or domes) exist in the B-T phase diagram of UTe_2. If the two superconducting states are unconventional, then disorder would suppress the superconducting transition temperature Tc and eventually destroy superconductivity while the mean free path is short enough. This may apply to both the low-field and high-field superconducting states of UTe_2. Considering that the low-field state has a lower Tc, it is likely that this superconducting state could be suppressed more quickly than the high-field state upon increasing disorder. On the other hand, it is also possible that the low-field state itself is more sensitive to disorder. Therefore, the findings of this manuscript can be naturally understood if we consider the effect of disorder on the pairing breaking of the two superconducting states. In fact, similar scenarios have been discussed in other heavy fermion compounds under pressure.

Second, with inclusion of the new data in the revised manuscript, this effect becomes more obvious if we look at the phase diagrams shown in Fig.2b and Fig.3. For example, one can see that both the low-field and the high-field states show an obvious suppression of Tc by reducing the RRR ratio, but likely the Tc in the low-field state is suppressed more quickly and therefore superconductivity is absent at zero field for the sample with RRR=7. I would suggest the authors to include this as a possible scenario in the discussion. If possible, it would be also nice to estimate and then compare the mean free path and the coherence length for samples with various RRR ratio, which may shed new insight into the superconducting state.

In summary, the revised manuscript has been largely improved and I can recommend it for publication in Nature Communications after some minor revisions.

Reviewer #3 (Remarks to the Author):

The authors have significantly improved the paper in response to earlier criticisms, which leads me to recommend its publication.

DEPARTMENT OF PHYSICS
Quantum Materials Center
John S. Toll Physics Building
4150 Campus Drive
College Park, MD 20742
Ofc: (301) 405 - 6143

Reviewer 1:

"This article reports on the occurrence of high-field superconductivity in a sample of UTe_2 in which no low-field superconductivity occurs. I have read the new version of the paper and saw that the authors have improved it compared to the previous version.

"The methodology used to obtain the experimental results is sound, the experimental findings are convincing, and the data analysis is carefully done. All of this is according to the standards in the field."

Author Response: Thank you for the previous comments which were helpful to the revision process.

"I now think that the paper has higher quality than before, but I am not sure it is worth a publication in Nature Communications. The finding to me isn't so surprising and new, and it doesn't directly lead to fundamentally new possible interpretations/insight. The authors claim that only this finding reveals that the high-field phase is of different origin than the low-field phase. However, this is not true. Other scenarios have been considered before (and are discussed and cited in this paper as well) and even the low-field phase diagram consists of two separate phases of possibly different origin."

Author Response: We respectfully disagree with the Reviewer's perspective. In fact, we believe that recent investigations into the stability of SC_2 in "next-generation" T_c = 2 K samples serve as another precedent which our results refute. In these recent reports, SC_2 appears far more fragile in the face of impurity scattering than SC_1.[1] This aligns with expectations established by previously reported reentrant superconducting phases in unconventional systems such as URhGe, which we discuss in the main text of our Manuscript.

Our observation of orphan superconductivity in UTe_2 stands in stark contrast to experimental precedent, including precent recently set by UTe_2 itself.[1] Our report confirms SC_{FP} to be *far more* robust than either SC_1 *or* SC_2, which refutes the theoretical assertion that "re-entrant superconductivity can be expected only if there is a superconducting transition in zero field… [because the] maximum transition temperature in high fields is predicted to be of the same order of magnitude as the zero field transition."[2]

[1] Wu, Z., Weinberger, T. I., Chen, J., Cabala, A., Chichinadze, D. V., Shaffer, D., ... & Eaton, A. G. (2023). Enhanced triplet superconductivity in next generation ultraclean UTe_2. *arXiv preprint arXiv:2305.19033*.

[2] Rieck, C. T., Scharnberg, K. & Klemm, R. A. Re-entrant superconductivity due to Landau level quantization? *Physica C: Superconductivity* **170**, 195-210, doi: 10.1016/0921-4534(90)90311-2, (1990).

UNIVERSITY OF MARYLAND 1856
DEPARTMENT OF PHYSICS

DEPARTMENT OF PHYSICS
Quantum Materials Center
John S. Toll Physics Building
4150 Campus Drive
College Park, MD 20742
Ofc: (301) 405 - 6143

"On the other hand I agree with the authors that this is the first finding of this kind and that the complicated phase diagram of UTe2 still lacks out theoretical understanding. But this was the case before as well."

Author Response: We wholeheartedly agree with the Reviewer as to the novelty of these results. We cannot overstate the importance of novel experimental results to the grounding of robust theoretical models. This is especially true when there are few relevant examples, as is the case in the field of reentrant and very high-field superconductivity. We hope, therefore, that our report will be of some use to our theoretical colleagues as well as to other experimentalists.

I also have more specific comments/requests of improvement.

Introduction

The introduction is quite lengthy and has a number of repetitions. I suggest the authors to write a sharper version, more to the point.

Author Response: As suggested, we have made several changes to "punch up" the Introduction. These have been highlighted in the revised Manuscript.

References

Some of the references should be updated. For example ref 16 which is now published, and 26/27, which are actually one publication with a new title and arxiv version published in Nat. Com.

In the last sentence of the first paragraph in the Results and Discussion section ref. 29 should be cited.

Author Response: We thank the Reviewer for these observations and have made the appropriate changes.

The discussion on RRR lacks giving the RRR of the main sample in this study in the text. This information is only given in fig. 4 a.

Author Response: We thank the Reviewer for this observation and have made the appropriate change, which has been highlighted in the Manuscript.

Quantum Materials Center
John S. Toll Physics Building
4150 Campus Drive
College Park, MD 20742
Ofc: (301) 405 - 6143

The discussion on Quantum-limit induced superconductivity lacks giving numbers of the Quantum limit fields for UTe2 which are simply given by the quantum oscillation frequencies. All of these frequencies are above 100 T, even for the disputed small 3D Fermi surface. These fields are far above the fields of field-induced SC in UTe2 and make this scenario unlikely. This should be mentioned.

Author Response: We have added an explicit reference to the quantum limit field as indicated by quantum oscillation measurements, and have highlighted this change in the main text.

Figures

Not much care is put into the figures. At some places, things that should be aligned (like axis labels of the same kind in different panels) are not, things that should have the same size, don't have it, etc.

Fig. 2 a and b have way too large labels. It actually reduces readability. Colors of text in the different phases in Fig. 2b are not well chosen (not readable). Point sizes are enormous.

Labels in Fig. 3 are also too big. Same for fig. 4.

Author Response: We thank the Reviewer for these observations regarding readability of the figures. We have adjusted Figs 1-3 in order to normalize axis thickness, text size, etc. We have also supplied all figure files to *Nature Communications'* copy editors as eps files without flattening.

DEPARTMENT OF PHYSICS
Quantum Materials Center
John S. Toll Physics Building
4150 Campus Drive
College Park, MD 20742
Ofc: (301) 405 - 6143

Reviewer 2:

"I have carefully read through the revised manuscript and the responses by the authors Corey E. Frank et al. The revised manuscript added new data with RRR=8.8 and updated the phase diagrams for comparison (revised Fig. 2b and Fig. 3b). Below are my comments and responses to the revised manuscript and the authors' rebuttal letter."

Author Response: Thank you for the previous comments, which were constructive.

"First, I'm afraid that the authors didn't carefully read the contents of my report and misunderstood its main points. Below is what I was trying to address in the initial report to Nature Physics: In literature, it has been reported that two superconducting states (or domes) exist in the B-T phase diagram of UTe_2. If the two superconducting states are unconventional, then disorder would suppress the superconducting transition temperature Tc and eventually destroy superconductivity while the mean free path is short enough. This may apply to both the low-field and high-field superconducting states of UTe_2."

Author Response: We agree with the Reviewer that the phase diagram of UTe_2 is complex, including multiple superconducting phases, and discuss these in the main text of our Manuscript. Likewise, we understand that unconventional superconductivity is readily suppressed by disorder, an effect which is observed in UTe_2 at both high and low fields.

"Considering that the low-field state has a lower Tc, it is likely that this superconducting state could be suppressed more quickly than the high-field state upon increasing disorder. On the other hand, it is also possible that the low-field state itself is more sensitive to disorder. Therefore, the findings of this manuscript can be naturally understood if we consider the effect of disorder on the pairing breaking of the two superconducting states. In fact, similar scenarios have been discussed in other heavy fermion compounds under pressure."

Author Response: We agree that our results suggest that the low-field state is more sensitive to disorder than the high field state, but respectfully disagree with the Reviewer's perspective that this finding is "natural." Indeed, the novelty of our result is the unprecedented sensitivity of SC_1 when compared to oSC_{FP}. We highlight in our Manuscript the case of URhGe, a heavy fermion reentrant superconductor in which the superconducting phase diagram has been previously compared between samples of different purities as defined by RRR. In this case, the reentrant phase follows the common expectation and is slightly more fragile than its low field parent, and thus

DEPARTMENT OF PHYSICS
Quantum Materials Center
John S. Toll Physics Building
4150 Campus Drive
College Park, MD 20742
Ofc: (301) 405 - 6143

experimental precedent could lead one to assume *a priori* that SC_{FP} would destabilize as-or-more quickly as a function of RRR than SC_1, as SC_2 appears[3] to do.

The Reviewer's reference to cases discussed only under the application of pressure further highlights the lack of relevant examples against which to compare our results. Due to the subtle and manifold effects of pressure on the already complex phase diagrams of heavy fermion superconductors generally, and UTe_2[4] specifically, such cases are beyond the scope of this report.

"Second, with inclusion of the new data in the revised manuscript, this effect becomes more obvious if we look at the phase diagrams shown in Fig.2b and Fig.3. For example, one can see that both the low-field and the high-field states show an obvious suppression of Tc by reducing the RRR ratio, but likely the Tc in the low-field state is suppressed more quickly and therefore superconductivity is absent at zero field for the sample with RRR=7. I would suggest the authors to include this as a possible scenario in the discussion."

Author Response: We agree with the Reviewer that Fig. 2b and Fig. 3 effectively communicate the suppression of T_c in UTe_2 with RRR, at least on the order-of-magnitude scale, and do not disagree that our results indicate SC_1 to be more fragile than oSC_{FP}. This observation in and of itself, in addition to the striking magnitude of the difference between SC_1 and oSC_{FP}, is the primary motivation for the Manuscript. In light of this comment, we agree that we did not previously articulate this finding sufficiently, and have made several changes to the Introduction in order to more effectively communicate the "thesis statement" of the work. These changes have been highlighted in yellow.

"If possible, it would be also nice to estimate and then compare the mean free path and the coherence length for samples with various RRR ratio, which may shed new insight into the superconducting state."

Author Response: We hesitate to follow this suggestion at this time as unfortunately any estimates of mean free path would be unreliable due to the unknown electronic structure. Moreover, we present in this work two samples with the same superconducting Tc (1.85 K) but very different residual resistance ratios (RRR = 8 and 64, respectively). This atypically large difference in RRR between the two relatively high-T_c samples strongly underscores the unreliability of resistivity as an indicator of pair-breaking interactions.

"In summary, the revised manuscript has been largely improved and I can recommend it for publication in Nature Communications after some minor revisions."

Author Response: We once again thank the Reviewer for their comments and suggestions throughout the revision process

[3] Wu, Z., Weinberger, T. I., Chen, J., Cabala, A., Chichinadze, D. V., Shaffer, D., ... & Eaton, A. G. (2023). Enhanced triplet superconductivity in next generation ultraclean UTe2. *arXiv preprint arXiv:2305.19033*.
[4] Sylvia K Lewin *et al* 2023 *Rep. Prog. Phys.* **86** 114501

DEPARTMENT OF PHYSICS

Quantum Materials Center
John S. Toll Physics Building
4150 Campus Drive
College Park, MD 20742
Ofc: (301) 405 - 6143

Reviewer 3:

"The authors have significantly improved the paper in response to earlier criticisms, which leads me to recommend its publication."

Author Response: We thank the Reviewer for their previous suggestions, which greatly assisted in the improvements which were made to the Manuscript.
